# Refractory Hyperkinetic Biliary Colic: A Case of Biliary Dyskinesia

**DOI:** 10.7759/cureus.102226

**Published:** 2026-01-24

**Authors:** Brielle Coe, Iman Elkhashab, Mian Arsam Haroon, Charles N Frasso

**Affiliations:** 1 College of Medicine, Drexel University College of Medicine, Philadelphia, USA; 2 Surgery, Philadelphia College of Osteopathic Medicine, Philadelphia, USA; 3 Surgery, Bayhealth Medical Center, Dover, USA; 4 Surgery, Bayhealth Medical Center, Milford, USA

**Keywords:** biliary dyskinesia, cholecystectomy, gall bladder dyskinesia, hida, right upper quadrant abdominal pain

## Abstract

Biliary dyskinesia results from abnormal gallbladder motility, leading to a deviation from a physiologic biliary ejection fraction. It is classified into two types: hyperkinetic and hypokinetic. Hyperkinetic biliary dyskinesia is an uncommon condition that may present without symptoms. In clinical practice, some patients are noted to have elevated gallbladder ejection fractions, though only a minority are considered for intervention. Due to its rarity and variable presentation, there are currently no established guidelines for treatment.

We present the case of a 48-year-old female with a year-long history of postprandial right upper quadrant (RUQ) pain, nausea, and weight loss, ultimately diagnosed with hyperkinetic biliary colic. Following a comprehensive evaluation, which included unremarkable imaging and a hepatobiliary iminodiacetic acid (HIDA) scan demonstrating 100% gallbladder ejection. In the following days, she underwent robotic-assisted cholecystectomy (RAC) with subsequent symptomatic improvement. This case highlights the diagnostic challenges, management considerations, and postoperative outcomes based on the detailed personal account in this rare clinical entity.

## Introduction

Gallbladder dyskinesia is a functional motility disorder of the gallbladder, leading to biliary colic and other gastrointestinal symptoms. While biliary dyskinesia is most often associated with hypokinetic dysfunction, characterized by a gallbladder ejection fraction (EF) of less than 35%, hyperkinetic biliary dyskinesia (HBD), marked by excessive gallbladder contraction, is a rare and less well-understood clinical finding that does not have clear management guidelines [1]. Although cholecystectomy is a well-established treatment for hypokinetic biliary dyskinesia, the role of surgical intervention in HBD remains controversial, with limited consensus on the appropriateness of surgical management and paucity of data in the literature.

Management strategies and symptom profiles for HBD are poorly characterized, making diagnosis challenging and likely contributing to underdiagnosis. HBD is typically defined by a gallbladder EF greater than 80% in the presence of biliary colic symptoms that may include intermittent and/or postprandial right upper quadrant (RUQ) abdominal pain, nausea, vomiting, bloating or early satiety, and diarrhea [2]. In one case series, nearly 70% of patients with elevated EF values developed symptoms, although the formal diagnostic criterion remains an EF exceeding 80% [3].

The pathophysiology of HBD is incompletely understood but is hypothesized to involve increased cholecystokinin (CCK) production, leading to more frequent and forceful gallbladder contractions [4]. The rapid emptying of the gallbladder may cause postprandial nausea and RUQ pain, while elevated cystic duct pressures may result in mucosal damage, sphincter of Oddi dysfunction, or even cholecystitis [5]. The demographic profile of HBD parallels that of hypokinetic disease, with middle-aged females being the predominantly affected cohort.

While prior studies, such as the case series by Camacho et al. [2], have demonstrated favorable outcomes following cholecystectomy for hyperkinetic biliary dyskinesia, detailed individual case reports remain limited. Therefore, this report contributes to the literature by providing an in-depth account of the diagnostic challenges, clinical decision-making process, and postoperative course in a patient with hyperkinetic biliary colic, highlighting the nuances involved in managing this poorly understood condition.

## Case presentation

Patient information

A 48-year-old female presented with a twelve-month history of intermittent, colicky RUQ pain. Her medical history included cesarean section (2014), tonsillectomy, cervical spine fusion, acute diverticulitis (2021), and irritable bowel syndrome (IBS). She denied any family history of gallbladder or gastrointestinal disorders. The patient was a lifelong nonsmoker, did not consume alcohol, and reported occasional cannabis use.

Primary concerns and symptoms

The patient reported sharp RUQ pain occurring approximately 20 minutes after meals, particularly after ingestion of fatty foods. The pain radiated to the back, was rated 10/10 at its worst, and was frequently associated with belching, postprandial nausea, vomiting, constipation, loss of appetite, and an unintentional 7-pound weight loss over the past six weeks prior to admission. She also endorsed persistent pruritus but denied fever, chills, dysuria, or respiratory symptoms.

Relevant past interventions and outcomes

A colonoscopy performed in 2021 for IBS symptoms was unremarkable. Patient was reportedly following up with a gastroenterologist, though no documentation of these encounters was able to be recovered.

Clinical findings

On physical examination, the patient appeared alert and oriented. Vital signs were stable. Abdominal examination revealed a flat, soft abdomen with focal tenderness in the RUQ and positive Murphy’s sign. The remainder of the examination was unremarkable. 

Diagnostic assessment

Initial laboratory testing, including bilirubin (0.6 mg/dL), liver function tests, complete blood count, and lipase, was within normal limits, ruling out biliary obstruction, infection, and pancreatitis (Table 1).

**Table 1:** Initial encounter laboratory values

Laboratory test	Value	Reference range
Total bilirubin	0.7	0-1.2 mg/dL
Aspartate aminotransferase (AST)	22	0-40 IU/L
Alanine aminotransferase (ALT)	17	0-32 IU/L
Lipase	51	23-300 units/L
WBC	4.6 x 10^3^	3.4-10.8 x 10^3^/uL
Hemoglobin	12.4	11.1-15.9 g/dL
Platelets	286 x 10^3^	150-140 x 10^3^/uL

**Figure 1:**
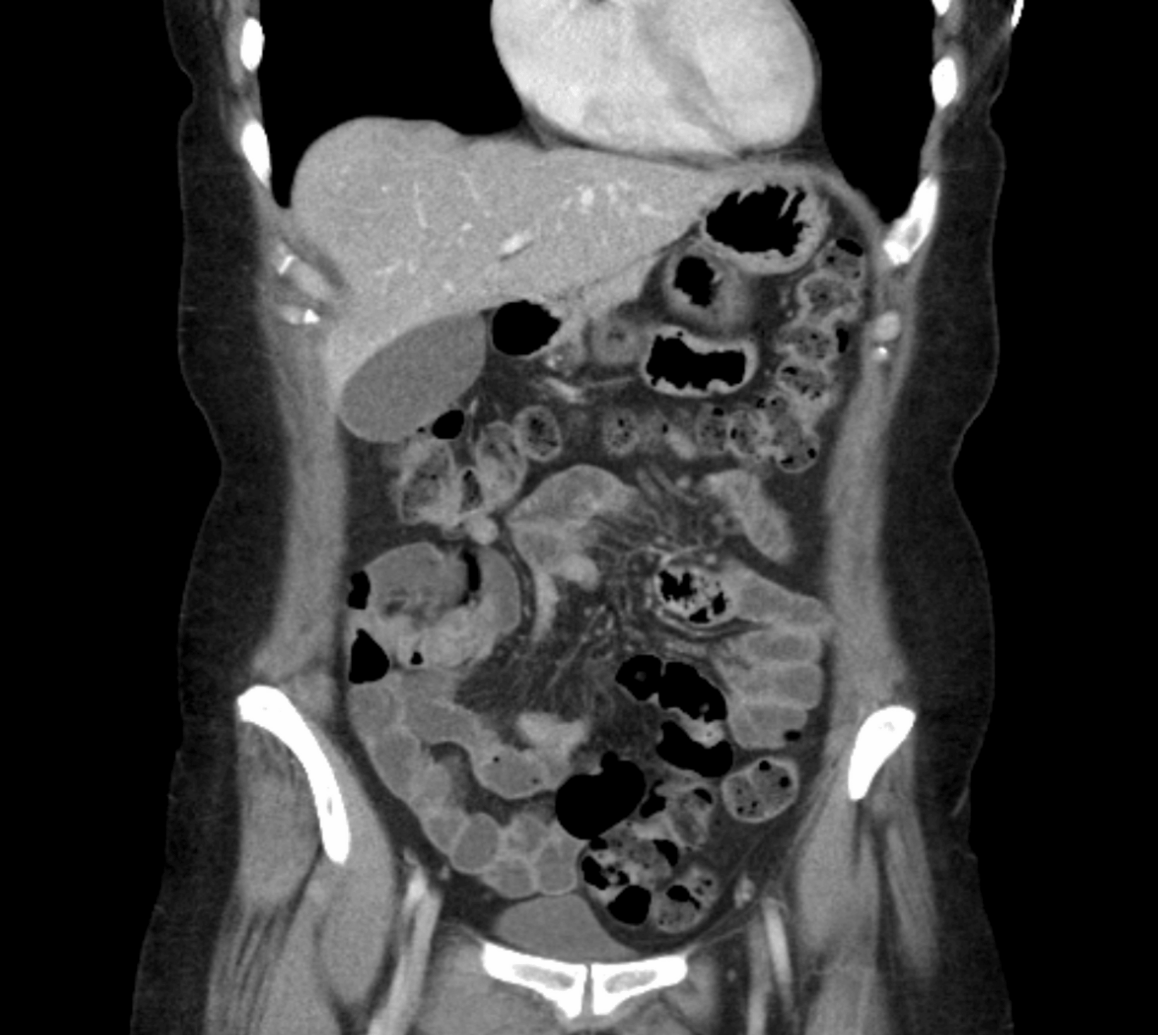
Coronal view of contrast-enhanced CT abdomen pelvis with gallbladder shown

Contrast-enhanced CT of the abdomen demonstrated a normal gallbladder without stones, wall thickening, or biliary ductal dilation (Figure 1). 

**Figure 2:**
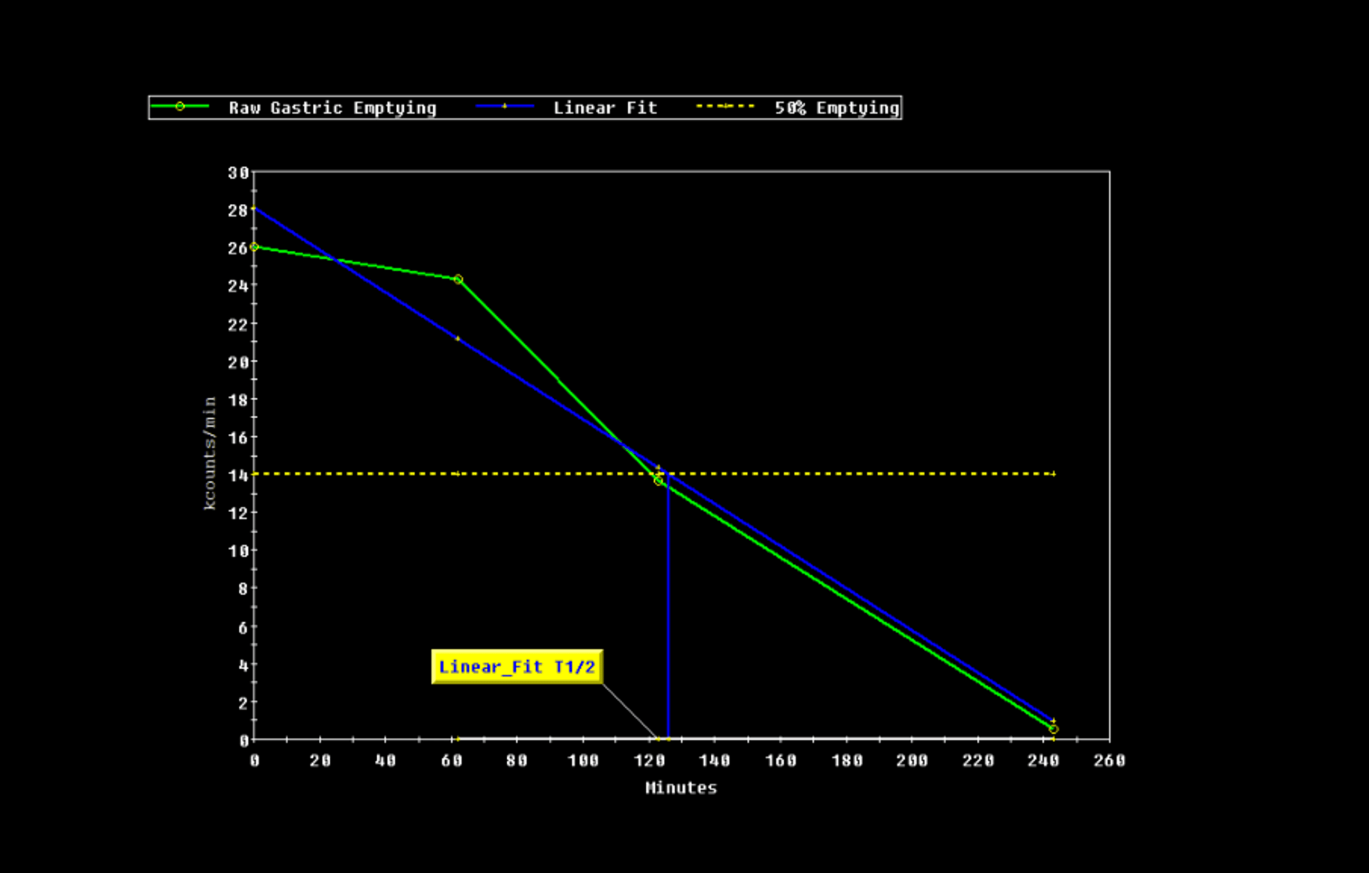
HIDA scan indicating 98% gallbladder emptying at 4 hours HIDA: hepatobiliary iminodiacetic acid

Given the persistent symptoms despite normal imaging and labs, a hepatobiliary iminodiacetic acid (HIDA) scan with CCK stimulation was performed, demonstrating an ejection fraction of 98%, consistent with hyperkinetic biliary dyskinesia (Figure 2). 

Diagnostic challenges included the absence of structural abnormalities on imaging and the nonspecific nature of her gastrointestinal symptoms. Differential diagnoses considered included functional dyspepsia, gastroesophageal reflux disease (GERD), sphincter of Oddi dysfunction, and irritable bowel syndrome, which ultimately were able to be meaningfully excluded.

Therapeutic intervention

Following counseling on the rare nature of her diagnosis and the uncertain benefit of surgical intervention, the patient elected to proceed with robotic-assisted cholecystectomy. The surgery was performed in December 2024 without intraoperative or immediate postoperative complications. Surgical pathology of the removed tissue demonstrated an 8.5 x 3.0 x 3.0 cm intact gallbladder with smooth, glistening, green serosa, with a patent cystic duct. The specimen's interior revealed a bile-stained velvety mucosa with diffuse yellow stippling; the lumen contained abundant dark viscous bile. The gallbladder wall thickness averaged 0.1 cm. No calculi were present.

Postoperatively, the patient received PRN (as needed) oxycodone for pain management and stool softeners for bowel regulation. Due to persistent nausea thought to be secondary to GERD, she was started on omeprazole.

Follow-up and outcomes

On postoperative day one, the patient reported mild incisional pain, was tolerating oral intake, and ambulated independently. She was discharged in stable condition. At her first postoperative follow-up on December 12, 2024, she reported marked improvement in nausea and abdominal pain. Physical examination revealed well-healed surgical incisions without signs of infection.

At a subsequent follow-up on January 9, 2025, she remained asymptomatic with no recurrence of abdominal pain. Mild constipation persisted but was effectively managed with stool softeners and laxatives. Her nausea at the time was attributed to underlying GERD rather than biliary pathology. No adverse or unanticipated events were reported during her postoperative course.

## Discussion

This case highlights the diagnostic and management challenges associated with hyperkinetic biliary dyskinesia (HBD), a rare and underrecognized cause of biliary colic. Our approach included the methodical exclusion of more common etiologies of RUQ pain through comprehensive laboratory testing and structural imaging, ensuring that the diagnosis of HBD was one of true exclusion in addition to meeting appropriate diagnostic criteria. Functional testing with a HIDA scan was employed after anatomic abnormalities were ruled out, which aligns with the diagnostic strategy recommended in the limited available literature [2].

However, limitations remain, including the inherent uncertainty regarding the natural history of HBD and the unpredictable outcomes following surgical intervention. Although the patient experienced significant symptomatic relief, the absence of standardized diagnostic criteria and the limited number of prospective studies mean that results may not be generalizable [4]. Additionally, the persistence of postoperative nausea, ultimately attributed to GERD, had briefly complicated the interpretation of early postoperative symptom resolution.

The existing medical literature on HBD remains sparse. Case series and retrospective analyses suggest that patients with high gallbladder ejection fractions and biliary-type pain may benefit from cholecystectomy. In one series by Huckaby et al., adolescents with biliary hyperkinesia achieved good postoperative outcomes following cholecystectomy, suggesting that surgical intervention can be beneficial when symptoms are severe and other causes are excluded [3]. Similarly, Camacho et al. (2024) [2] and Kartik et al. (2023) [4] reported that a high proportion of adults undergoing cholecystectomy for HBD experienced postoperative improvement. However, the exact pathophysiologic mechanisms remain speculative, with hypotheses including heightened sensitivity to cholecystokinin, high-pressure emptying causing cystic duct injury, and sphincter of Oddi dysfunction [5]. These gaps in understanding underscore the need for prospective, multicenter studies to better define diagnostic criteria and management strategies.

In our case, the decision to proceed with cholecystectomy was driven by the severity of the patient's symptoms, the exclusion of structural or infectious causes, the confirmation of hyperkinetic function on HIDA scan, and informed discussion of the uncertain but potentially favorable outcomes. The patient's subsequent symptomatic improvement provides additional support for considering cholecystectomy in select patients with persistent hyperkinetic biliary dyskinesia-related symptoms, although careful patient counseling remains paramount, emphasizing that surgical intervention has been successful in some cases but is not current guideline practice.

## Conclusions

Hyperkinetic biliary dyskinesia is an uncommon but important cause of biliary colic that should be considered in patients presenting with characteristic symptoms, unremarkable structural imaging, and medically refractory disease. Functional testing with a HIDA scan is crucial for diagnosis. Cholecystectomy may offer significant symptomatic relief, but careful patient selection and thorough preoperative counseling are essential. This case underscores the importance of maintaining a broad differential diagnosis and highlights the need for individualized, patient-centered decision-making when managing rare functional gallbladder disorders.
